# Infective Endocarditis in Disseminated *Streptococcus dysgalactiae* Infection Originating From Hitherto Undetected Colorectal Adenocarcinoma

**DOI:** 10.1155/crdi/4134020

**Published:** 2026-04-08

**Authors:** Roel Meeus, Lander Bacquaert, Filip Baert, Karl Dujardin, Dirk Vogelaers

**Affiliations:** ^1^ Department of Gastro-Enterology, AZ Delta, Roeselare, Belgium; ^2^ Department of Cardiology, AZ Delta, Roeselare, Belgium; ^3^ Department of Infectious Diseases, AZ Delta, Roeselare, Belgium; ^4^ Department of Internal Medicine, Universitair Ziekenhuis Gent, Ghent, Belgium, uzgent.be

**Keywords:** brain emboli, colonoscopy, infective endocarditis, rectal adenocarcinoma, streptococcus

## Abstract

A 60‐year‐old woman presented with polyarthritis in the aftermath of an upper respiratory tract infection. Further investigation revealed infective mitral valve endocarditis necessitating acute surgical valve replacement due to *Streptococcus dysgalactiae*, as evidenced by positive blood and intraoperative cultures, with disseminated infection and septic emboli in kidneys, multiple joints, and brain. Subsequent colonoscopy revealed a hitherto undetected rectal adenocarcinoma. The co‐occurrence of *S. dysgalactiae* endocarditis and colorectal malignancy in this patient, who was within the age range of elevated baseline risk, may represent coincidental coexistence rather than a causal relationship. This case adds to the sparse literature linking *S. dysgalactiae* infection with colorectal neoplasia and suggests that colonoscopy may be considered on an individualized, risk‐adapted basis in selected patients with *S. dysgalactiae* endocarditis—an approach distinct from the evidence‐based colonoscopy recommendations applicable to *Streptococcus gallolyticus* endocarditis.

## 1. Introduction

Infective endocarditis (IE) poses a significant clinical challenge due to its complexity and potentially life‐threatening course. While *Streptococcus viridans* is a classic endocarditis‐prone pathogen, particularly in subacute IE, the role of *Streptococcus dysgalactiae*—a Group C or G β‐hemolytic streptococcus—remains relatively underexplored. Recent trends, however, suggest a concerning rise in *S. dysgalactiae*‐associated bacteremia and endocarditis. This organism has been increasingly recognized for its aggressive clinical behavior, including rapid tissue invasion, embolic complications, and associations with underlying comorbidities [1, 2].

The well‐established association between *Streptococcus gallolyticus* (formerly *Streptococcus bovis*) and colorectal neoplasia has prompted guideline‐endorsed colonoscopy screening in patients with *S. gallolyticus* endocarditis. Whether an analogous relationship exists for *S. dysgalactiae* remains uncertain, with only sparse case‐based evidence linking *S. dysgalactiae* infection to underlying colorectal malignancy [3, 4]. Here, we report a case of *S. dysgalactiae* IE with disseminated infection in which colonoscopy revealed a previously undetected rectal adenocarcinoma, adding to the sparse but growing body of literature on this potential association.

## 2. Case Presentation

**FIGURE 1:**
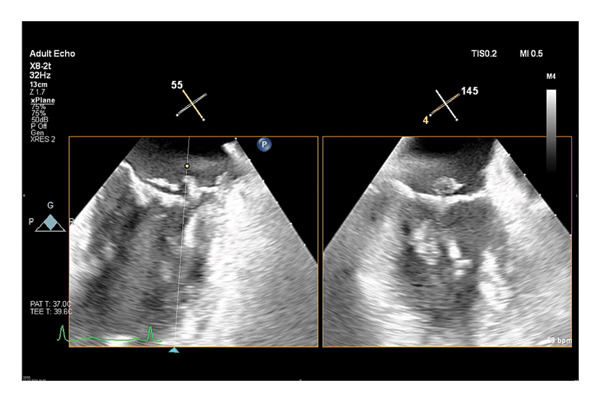
Endocarditis with vegetations on the atrial side of the mitral valve, flail leaflet in the anterolateral segment P1 of the posterior mitral valve.

A 60‐year‐old woman was admitted to the hospital with polyarthritis, following an upper respiratory tract infection three weeks before, characterized by rhinitis without fever or throat pain. Physical examination revealed swelling and redness in the left thenar eminence and wrist, right thenar eminence, both knees, left first metatarsal head, and right tarsals. Laboratory tests indicated a creatinine level of 1.22 mg/dL and a CRP level of 343 mg/L, along with slightly aberrant liver function tests and a platelet count of 78,000 × 10^3^/µL. Abdominal CT and ultrasound showed hepatomegaly with steatosis without further abnormalities. Blood cultures were drawn, and synovial fluid analysis revealed an inflammatory profile with a leukocyte count of 4,539/μL (62% neutrophils); cultures remained negative. A transesophageal echocardiography (TEE) revealed normal systolic and diastolic function, a vegetation on the mitral valve, and mitral insufficiency graded 1‐2/4 (Figure 1). The patient was diagnosed with disseminated *S. dysgalactiae* infection including septic polyarthritis and mitral valve endocarditis and was initially treated with high‐dose penicillin (24 million units/24 h).

On Day 2, the patient underwent primary mitral valve replacement with implantation of a 31‐mm mechanical bileaflet prosthetic valve (SJM Masters Series). The decision for early surgical intervention was based on the presence of a large, mobile vegetation with evidence of ongoing systemic embolization, in accordance with current guideline recommendations for surgery in the active phase of IE, despite only mild mitral regurgitation on TEE. Cultures of the native mitral valve proved positive for *S. dysgalactiae*, leading to the addition of gentamicin and high‐dose amoxicillin.

On Day 4, acute kidney failure developed with a peak serum creatinine of 4.44 mg/dL, necessitating temporary dialysis and the interruption of gentamycin. Diuresis was restored, leading to rapid recovery of renal function. Gentamycin was subsequently restarted without repercussions on kidney function. Follow‐up CT showed bilateral multiple kidney infarctions.

**FIGURE 2:**
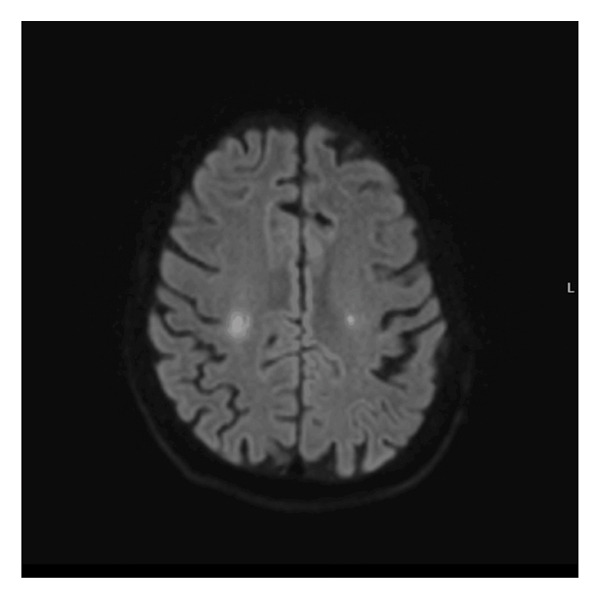
MRI of the brain with ischemic lesions consistent with septic emboli and microabscesses bilaterally in the semioval center.

On Day 6, a repeat arthroscopic lavage of both knees was performed due to ongoing septic arthritis and rising CRP levels. Clinical recurrence of septic arthritis in the left knee led to a second arthroscopic lavage, a couple of days later. During the following week, persistent weakness in the left arm prompted an MRI of the brain, revealing ischemic lesions consistent with septic emboli and microabscesses bilaterally in the semioval center (Figure 2).

After ICU transfer, rectal bleeding occurred, initially attributed to anticoagulation after mitral valve replacement. Abdominal MRI ruled out microaneurysms associated with *S. dysgalactiae* endocarditis, with sigmoidoscopy revealing an irregular necrotizing mass 7 cm above the anal sphincter, with histopathologic confirmation of rectal adenocarcinoma without microsatellite instability.

Further tumor staging showed bilateral pleural fluid on CT thorax and bilateral multiple kidney infarctions but no signs of metastasis. MRI staging indicated cT3cN1bM0 with invasion into the internal anal sphincter (Rullier type III) and narrow proximity of the external anal sphincter (Rullier type IV). The patient was started on neoadjuvant therapy according to the “PRODIGE regimen” with 12 weeks of FOLFIRINOX light (6 cycles), followed by 5 weeks of chemoradiotherapy with oral capecitabine, initially given under IV amoxicillin/clavulanic acid coverage.

A few days after staging, the patient complained of vaginal blood clots, and a rectovaginal fistula was confirmed by the gynecologist. Staging was subsequently revised to cT4N1bM0, reflecting direct invasion of the colorectal tumor into adjacent vaginal tissue.

## 3. Discussion

In recent years, there is been a notable shift in understanding Group G Streptococcus (GGS), previously regarded mainly as animal pathogens, now recognized for their impact on human health. GGS commonly colonizes various human anatomical sites like the skin, oropharynx, and female genital tract, facilitated by its morphological resemblance to Group A Streptococcus. The presence of the M‐associated protein enhances GGS’s clinical potency by evading immune recognition. Clinically, GGS shares similarities with Group A Streptococcus (GABHS), causing skin and throat infections, and can lead to severe bloodstream infections like endocarditis, pneumonia, and meningitis, akin to *Streptococcus pyogenes*. Predisposing factors for GGS‐related bloodstream infections include immune suppression, alcoholism, diabetes mellitus, neurological disorders, cancer, and compromised skin integrity. Common routes of GGS infection include lower extremity cellulitis, pressure ulcers, and conditions leading to skin breakdown [1, 2].

Approximately 6% of patients with GGS bacteremia develop endocarditis, often occurring in individuals with unexplained bacteremia and underlying comorbidities. Mortality rates in GGS bacteremia are significant, emphasizing the severity of the condition [3]. The progression of GGS endocarditis tends to be acute, with a high likelihood of disseminated disease. Incidence of embolic complications is high, reaching up to 64%, occurring twice as frequently as in other forms of IE [3–5].

The increasing incidence of GGS endocarditis, particularly among individuals with predisposing factors, highlights the importance of understanding the clinical manifestations and disease course of GGS endocarditis for improved management and outcomes [3].

The association between *S. bovis* endocarditis and colorectal cancer has been extensively documented [6]. Identification of streptococci to the species level is imperative. For *S. bovis/S. gallolyticus* IE, colonoscopy is recommended, given the firmly established association with colorectal neoplasia; in cases where no tumor is detected, follow‐up colonoscopy is endorsed by guidelines. This approach is distinct from *S. dysgalactiae*, where no equivalent evidence base exists, and colonoscopy should, therefore, be considered on an individualized, risk‐adapted basis rather than as a universal recommendation [7, 8].

As such, colonoscopy remains indispensable in the context of *S. bovis* endocarditis, as emphasized in various case studies [6, 7, 9, 10]. Fluorodeoxyglucose (FDG) positron emission tomography/computed tomography (PET/CT) is increasingly employed in IE diagnosis and may aid in detecting a pathologic gastrointestinal focus, thus guiding subsequent colonoscopy. However, it is crucial to note that a negative PET/CT does not definitively exclude significant colonic pathology. Furthermore, the clinical utility of FDG PET/CT in detecting occult colorectal cancer in *S. bovis/S. gallolyticus* IE patients remains to be studied [8].

The association between GGS and colorectal cancer has been sparsely documented in the medical literature, with only a few case reports published to date [10]. While *S. dysgalactiae* can colonize various anatomical sites, including the colorectal region, conclusive evidence linking it to colorectal cancer is lacking. In the case report by Abugroun et al., a patient with GGS endocarditis was found to have colorectal carcinoma, highlighting a potential, albeit rare, link [10]. Beyond isolated case reports, older observational studies have also hinted at a broader association between GGS infections and underlying malignancies. In the literature, underlying malignancy has been reported in 21%–65% of the cases of GGS bacteremia [11, 12]. Malignancy has consistently emerged as one of the most significant underlying conditions associated with GGS infections, with reports suggesting that up to two‐thirds of affected patients may have an underlying cancer—even in the absence of metastasis or chemotherapy. Our case similarly demonstrates this association, but with several notable distinctions: our patient exhibited a highly aggressive clinical course, including systemic embolization to the brain, kidneys, and joints, acute kidney injury requiring temporary dialysis, repeated septic arthritis interventions, and ICU admission. This emphasizes that *S. dysgalactiae* endocarditis can be severe even in immunocompetent patients with no prior comorbidities, unlike many reported cases where host vulnerability may have contributed [13].

Speculation exists that colorectal adenocarcinoma may serve as a portal of entry for *S. dysgalactiae*, analogous to the established *S. bovis*–colorectal cancer relationship [3]. In our patient, the tumor likely disrupted mucosal integrity, facilitating bacterial translocation into the bloodstream. This aligns with the proposed pathophysiology in *S. bovis* infections, where tumors act as both microbial reservoirs and entry points due to local immune dysregulation and epithelial breach. Beyond mechanical disruption, chronic local inflammation and bacterial‐induced dysbiosis may create a tumor‐promoting microenvironment, further increasing susceptibility to invasive infection. Proinflammatory cytokines such as TNF‐α, often upregulated during bacterial infection, have been implicated in carcinogenesis, suggesting a possible interplay between bacterial colonization, systemic inflammation, and tumor biology [6, 14]. Clinically, the co‐occurrence of *S. dysgalactiae* endocarditis and colorectal adenocarcinoma in our patient—who fell within the age range of elevated baseline colorectal cancer risk—may represent coincidental coexistence rather than a causal relationship. Nonetheless, colonoscopy performed during the admission enabled early detection of the rectal adenocarcinoma, allowing timely initiation of neoadjuvant therapy. This approach—distinct from the evidence‐based colonoscopy recommendations for *S. gallolyticus* endocarditis, where the association with colorectal neoplasia is firmly established and guideline‐endorsed—should be considered on a risk‐adapted, individualized basis in patients with *S. dysgalactiae* endocarditis, particularly those who are older, have no identified infectious source, or present with disseminated infection.

In conclusion, although a causal relationship between *S. dysgalactiae* and colorectal cancer has not been established, the organism’s emerging role as an invasive human pathogen, its phylogenetic relatedness to *S. bovis*, and shared virulence factors with other bacteria previously linked to colorectal neoplasia warrant further investigation. While colorectal cancer is relatively common in older adults, a growing number of case reports, including our own, describe *S. dysgalactiae* infections in patients with underlying malignancies, suggesting that clinicians should remain alert when encountering *S. dysgalactiae* endocarditis or bacteremia without a clear primary source. In such cases, colonoscopic evaluation may be considered as part of a broader, risk‐adapted diagnostic workup, particularly in older adults or those with additional CRC risk factors. Universal screening cannot be recommended at this stage. Multidisciplinary collaboration between infectious disease specialists, gastroenterologists, and oncologists, along with well‐designed prospective studies, will be essential to clarify the clinical significance of this association and guide future recommendations.

## 4. Conclusions

In summary, this case underscores the increasing recognition of *S. dysgalactiae* as a cause of aggressive IE with disseminated septic emboli. The co‐occurrence of *S. dysgalactiae* endocarditis and colorectal adenocarcinoma in this patient may represent coincidental coexistence in an at‐risk age group rather than a causal relationship and should be clearly distinguished from the well‐established *S. gallolyticus*–colorectal neoplasia link. While available evidence does not support universal colonoscopy in *S. dysgalactiae* endocarditis, a risk‐adapted, individualized approach may be warranted in selected patients. Further prospective data are needed to clarify the clinical significance of this potential association [15].

## Author Contributions

All authors contributed to the conception and design. Material preparation and analysis were performed by Roel Meeus and Lander Bacquaert. The first draft of the manuscript was written by Roel Meeus and Lander Bacquaert and all authors commented on previous versions of the manuscript.

## Funding

The authors declare that no funds, grants, or other support were received during the preparation of this manuscript.

## Disclosure

All authors have read and approved the final manuscript.

## Ethics Statement

The authors confirm that the study was performed in accordance with the ethical standards if the institutional and/or national research committee and with the 1964 Helsinki Declaration and its later amendments or comparable ethical standards.

## Consent

The authors confirm that informed consent to participate was obtained from the individual included in the study.

Written informed consent was obtained for publication of this manuscript and the accompanying figures (Figures 1 and 2).

## Conflicts of Interest

Author Filip Baert declares grant support from AbbVie, Amgen, Janssen, and Takeda Honorarium and speakers fees from AbbVie, Amgen, Arena, Celltrion, Ferring Holding SA, Fresenius Kabi AG, Galapagos, Janssen, Merck Sharp & Dohme, Pfizer, and Takeda. All other authors declare no conflicts of interest.

## Data Availability

Data sharing is not applicable to this article as no datasets were generated or analyzed during the current study.
